# The Public Distribution System and Food Security in India

**DOI:** 10.3390/ijerph16173221

**Published:** 2019-09-03

**Authors:** Neetu Abey George, Fiona H. McKay

**Affiliations:** School of Health and Social Development, Faculty of Health, Deakin University, Melbourne Burwood Campus, 221 Burwood Highway, Burwood, VIC 3125, Australia

**Keywords:** India, food security, Public Distribution System, National Food Security Act

## Abstract

The Public Distribution System (PDS) of India plays a crucial role in reducing food insecurity by acting as a safety net by distributing essentials at a subsidised rate. While the PDS forms a cornerstone of government food and nutrition policy, India continues to be home to a large population of hungry and malnourished people. This review seeks to explore the functioning and efficiency of the PDS in achieving food and nutritional security in India. A comprehensive and systematic search using the key terms “food insecurity” OR “food security” AND “Public Distribution System” OR “PDS” OR “TPDS” AND “India” identified 23 articles which met the inclusion criteria. This review draws attention to the lack of published literature in areas of PDS and food security in India. The findings of the review emphasise the role of PDS in tackling hunger and malnutrition while highlighting its limited role in improving food security and childhood mortality due to operational inefficiencies. The PDS has the potential to act as a solution to food insecurity in India if the operational inefficiencies and environmental footprints are addressed by adequate policy reforms.

## 1. Introduction

Food insecurity is a situation of limited access to safe and healthy food [1], while food security refers to a situation when ‘all people, at all times, have physical, social, and economic access to sufficient, safe and nutritious food that meets their dietary needs and food preferences for an active and healthy life’ [2]. Food insecurity can be understood as a continuum that progresses from uncertainty and anxiety about access to sufficient and appropriate food at the household level, to the extreme condition of hunger among children because they do not have enough to eat [3]. The experience of food insecurity has been found to be more severe in low-income communities, and for those who already experience poor health [4,5]. While factors contributing to poverty are important when considering food insecurity, it is not the only determinant. Other influences include policy, the distribution of food across populations, countries, and regions, unstable political conditions, and climate change induced adverse environmental conditions including severe droughts, lack of water, and soil degradation and erosion [1,6,7,8,9]. 

## 2. Food Security in India

While overall global rates of food insecurity have decreased, there remains a large population of people experiencing food insecurity living in sub-Saharan African and South Asia. Countries most affected are typically characterised by high rates of disease and mortality associated with nutritional deficiencies coupled with high rates of poverty [10,11,12]. Despite rapid economic growth over the last two decades, many Indians have not benefited from the economic improvement, and continue to experience food insecurity and hunger, a high burden of malnutrition and undernourishment, and increasing obesity [13]; in 2016, over 190 million people were reported undernourished—the highest in any single country [14]. 

The reasons behind food insecurity and hunger in India are complex. Some research indicates that high rates of migration from rural to urban areas may play a role [15], as evidenced by the concentration of economic gains in urban areas, and the significant changes to the contribution of the agriculture sector to GDP [16]. The adverse effects of climate change are also an emerging contributor to food insecurity, with uneven weather patterns and increasing droughts to blame for uneven growth and production of food stock [5]. The most recent Global Hunger Index (GHI) ranks India at the high end of the ‘serious’ category, as India continues to perform poorly in addressing hunger and malnutrition; currently one in three Indian children is stunted representing one third of the world’s stunted population, and one in five is wasted [17]. Many in India also experiences hidden hunger. Hidden hunger refers to a situation of chronic micronutrient deficiency, where a person might have access to sufficient calories, but lacks adequate micronutrients [18]. Hidden hunger can have lasting effects on health and wellbeing, and is especially problematic for children [19]. 

The government of India have adopted a range of policies over the last 60 years in an attempt to strengthen food security [5]. One of the key responses to food insecurity and hunger in India is the distribution of food grains through the government controlled Public Distribution System (PDS) [20]. Established after World War Two with an aim of increasing domestic agricultural production and improving food security, the PDS has evolved to the largest universal distribution system in the world for the supply of subsidised food grains [20,21]. Through a partnership between central and state governments, the food-safety net program aims to supplement essential household supplies including wheat, rice, sugar, and kerosene. However, like other solutions to food scarcity, this program is not intended to provide all required household foods, but rather provide goods to supplement purchased or farmed goods [20,22]. To facilitate the distribution, the Food Corporation of India (FCI) acts a central nodal agency responsible for the procurement of food grains from farmers at a price that is often higher than market price [20,23]. The individual state governments then procure the food grains at a subsidised price known as the ‘central issue price’ from the FCI [20,24], these goods are then distributed to consumers via fair price or ration shops [20]. 

Amendments made to the PDS in June 1992 sought to improve coverage, especially to those living in disadvantaged, remote, or difficult to access areas [20]. The PDS was re-structured again in June 1997, to better target lower socio-economic areas [20]. This Targeted Public Distribution System (TPDS) aimed to provide over seven million tonnes of foods to 60 million households identified as below the poverty line [20]. This was followed by the introduction (in December 2000 and expansion in 2003–2006) of the Antyodaya Anna Yojana (a Hindi term meaning “grain scheme for the downtrodden” [25]) scheme to provide highly subsidised foods to India’s poor. The scheme was an attempt to streamline the PDS to more efficiently target the poorest of the poor. This expansion of the PDS also included provision of food and goods to senior citizens and pensioners over 60 years, as well as widows and people affected by disease or infirmity [20]. 

These measures to strengthen the PDS received statutory backing through the introduction of the National Food Security Act (NFSA) [20,26,27]. Through the adoption of a life cycle approach, the importance of food security was emphasised by the NFSA. Through its implementation, the PDS achieved 75% coverage of the rural population, and around half of the urban population, monetary and nutritional support was mandated to pregnant and lactating women, and through the Integrated Child Development Services and Mid-Day Meal Schemes, children aged 6 months to 14 years were also covered [20,26,28,29,30]. The NFSA marked an important milestone in that it awarded India’s food safety network a legal standing in accordance with the fundamental right to good health and nutritious food [20,26,31]. The NSFA also went some way to address the challenges faced by the TPDS in the form of corruption and diversion by enforcing more accountability on stakeholders while improving the transparency of its operation [21,28].

While a number of studies have identified the role of the PDS and NFSA in improving the food security of India, at least through the provision of calories [32,33], there is some concern relating to issues of miss-targeting, under-coverage, corruption and diversion affecting the implementation and operation of the food safety network in India [13,26,34]. Understanding these concerns is challenging, as while a number of non-government organisations and government bodies provide reports on the quantity of food distributed [35], and the number of people reached through the PDS [36,37], there is little analysis or evidence surrounding the broader issues related to food insecurity and the PDS. Furthermore, these reports rarely provide the method for data collection and/or analysis, making further interpretation difficult. This current review seeks to bring together the published literature on the PDS, in order to investigate the role it plays in addressing food insecurity in India. This review seeks to understand this large and expensive food distribution system and its role in one of the most populist, but inequitable countries. This is the first review of its kind of this program, with the objective of providing a clear overview of current knowledge in this area. By investigating the available peer-reviewed literature, this review seeks to understand the role of the PDS in any attempt to achieve food security in India.

Specifically, there are two main objectives to this review:

1. To investigate the role of the PDS in delivering an efficient food-safety network in India;
2. To investigate the barriers and enablers of the PDS.

## 3. Method and Approach

A systematic literature search was conducted in November 2018 to identify articles that investigated the role of the PDS in responding to food insecurity in India. Databases included; Academic Search Complete, CINAHL Complete, EconLit, Global Health, GreenFILE, Health Policy Reference Center, Legal Source, Scopus, and Medline. Search terms included “food insecurity” OR “food security” AND “Public Distribution System” OR “PDS” OR “TPDS” AND “India”. Limits restricted the search to those articles with full text published in English. In order to gain a comprehensive understanding of the role of the PDS in achieving food security over time, no temporal limitations were placed on articles. 

Articles were included in this review if they (1) included food security or food insecurity as an exposure of interest; (2) investigated the role of the PDS in addressing food security; and (3) were peer-reviewed. Editorials or commentaries [34,38,39,40,41] were excluded. Both authors reviewed all articles to identify relevancy. Articles were first screened by title and abstract based on the inclusion criteria. The full text of selected articles was obtained for assessment for final inclusion.

## 4. Results

**Figure 1:**
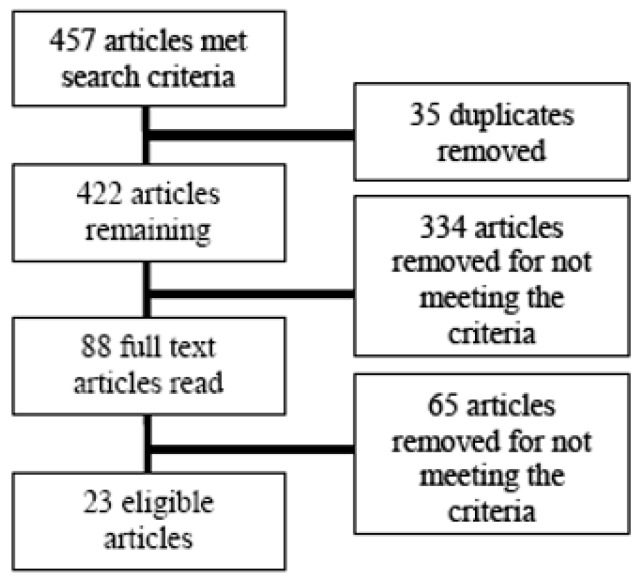
Flow chart showing article selection.

The database search utilising the key words identified 457 articles, of which 35 were duplicates. The titles and abstracts of the remaining 422 articles were reviewed to determine eligibility. Of these 334 articles were rejected based on the title or abstract, the full text of the remaining 88 articles was reviewed, leaving 23 that met the inclusion criteria (Figure 1). 

### 4.1. Study Characteristics

Reflecting recent interest in issues of the role of the PDS in addressing food insecurity, most articles (*n* = 19) were published between 2010 and 2018. While some (*n* = 11) articles reported on data collected by the National Sample Survey (NSS), India’s nation-wide household survey conducted on various socio-economic issues, only three articles reported on data that was collected at a national level [42,43,44]. The remaining studies focused on single states or a group of states, the most included states were Chhattisgarh, Andhra Pradesh, and Uttar Pradesh (see Table 1). 

Four studies employed mixed methods [45,46,47,48]. These studies employed a variety of methods including household interviews, and interviews with individual household members, surveys and interviews with PDS beneficiaries, and interviews with shop owners. Three studies were qualitative; Chopra, et al. [49] conducted interviews with 98 key informants including rice millers, production workers and shop owners; Dar [50] conducted interviews with 266 households in Kashmir to investigate a range of issues related to food access and entitlement; while Panigrahi and Pathak [51] conducted 50 interviews with above poverty line (APL) households and 50 interviews with below poverty line (BPL) households in Odisha to better understand their experiences with the PDS. All the remaining studies employed quantitative methods. Studies that focused on households ranged in participant household numbers from 50 [51] to more than half a million [44], while studies focused on individuals ranged in participant numbers from 98 [49] to 7124 [45]. 

### 4.2. Effectiveness of the PDS

Eight articles specifically examined the effectiveness of the PDS [45,46,47,49,53,54,56,58]. Each of these studies suggested that the PDS was not working effectively, with large amounts of food not reaching the intended recipients, and significant wastage resulting in high costs for limited benefits. For example, Dhanaraj and Gade [53] estimated that in Tamil Nadu, for every 5.43 kgs of PDS rice distributed by the government, only 1 kg reached those in need; the distribution was less efficient in the case of sugar, where only 1 kg for each 8.21 kgs distributed was consumed by those in need. Kumar [58], in a large investigation spanning 12 states, found that up to 100% of wheat was diverted in some cases, with diversion and provision of rice and wheat being different across all states. Khera [54], suggested that households cannot access their full entitlement to goods, and as a result are forced to purchase much of their food from the free market. Conversely, a positive trend was identified in the state of Bihar, where in 1993, 90% of food grains were diverted away from those in need; by 2001 this figure was down to just 12.5% of diverted food grain [56]. Similar findings were reported by Nair [61] in Kerala. In both states, this was attributed to better transparency and infrastructure.

### 4.3. Barriers and Enablers to the Efficient Working of the PDS

A number of barriers and enablers influencing the efficient working of the PDS were investigated across the studies included in this review. One key barrier to a more efficient system was the presence of illegal (or ghost) cards, with the finding that some households hold multiple cards [51,58]. The illegal cards were identified in several states, with Kumar [58] suggesting that there were approximately 230 million excess cards across the country in 2006. 

Despite a number of significant, system-wide changes over recent years, high levels of corruption and leakage continue to plague the PDS [48,53,54]. Part of this leakage occurs at the level of the fair price shops, where Gupta and Singh [48] reported that some store owners exchanged the high quality goods provided from the government for distribution through the PDS with lesser quality goods from the general stores. Both Khera [54] and Dhanaraj and Gade [53] reported very high rates of corruption within the system, in some states this was up to 100% leakage or ‘diversion’ from the supply chain. Transparency, better governance, technology and the introduction of computerisation, along with use of global positioning system and distribution via doorstep delivery have been suggested as potential ways to address these issues [45,56]. 

Targeting errors, specifically the problems associated with targeting BPL and APL households were identified as areas where efficiencies could be made [45,52,58]. These studies suggest that while there was some effort to target the BPL households, the targeting has had a marginal effect on poor households [52]. There is also a suggestion from Kumar [58] that non-poor households have been included in the PDS to the detriment of the system. This is consistent with the work of Nair [61], who suggest that better targeting and the removal of APL households, that is, a removal of universal nature of the system, would have significant positive impacts on the operation and effects of the PDS. 

### 4.4. Food Security in India—A Concept Map

**Figure 2:**
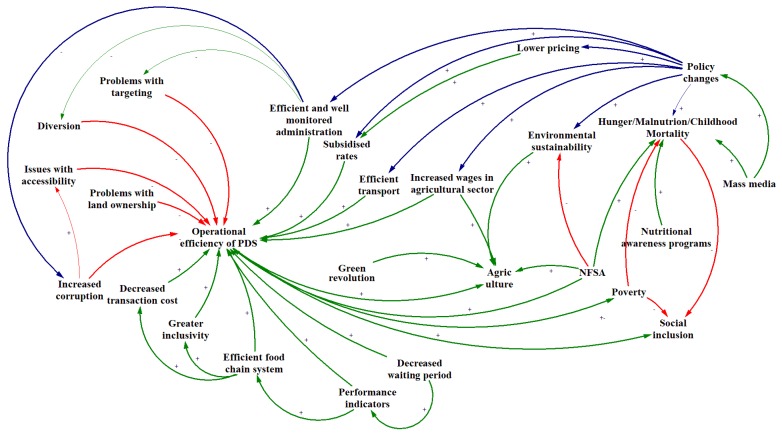
A concept of the various determinants that affect effectiveness of the PDS and NFSA in addressing the food security in India. NFSA: National Food Security Act; PDS: Public Distribution System.

In attempting to dissect the various areas that affect the effectiveness of the PDS and incorporate the involvement of multiple interrelated factors in addressing food security in India, a concept map was created (Figure 2). In the conceptual representation of the factors that determine the national food security, blue arrows signify those factors that can be influenced by policy changes. The red arrows and negative polarity indicate elements that act as barriers while the green arrows and positive polarity specify variables that act as enablers. 

As shown in Figure 2, there are multiple cross-links between the domains influencing food insecurity; the complex nature of the problem means that there is no one single overarching solution. One of the main responses to food insecurity in India, the PDS, is riddled with problems of targeting, diversion, and corruption [45,54,56]. As shown here, increased corruption decreases the accessibility which in turn affects the overall functioning of the PDS [54,56]. Despite the existence of multiple barriers, the PDS can be fortified if the enablers identified here are concentrated on. Efficient and well monitored administration was observed as a conclusive answer to not just problems with targeting but is also an effective solution for diversion [21,45,54,56]. Subsidised rates for commodities and an efficient transport system can also be drivers of success [21,45], as can an efficient food chain system that fosters greater inclusivity while reducing transaction costs [21,45]. Finally, increased wages in the agricultural sector were also recognised as a facilitating factor for the functioning of the PDS and agriculture in India [45,64].

The NFSA also plays a key role in responding to food insecurity. Having been a positive reinforcement for the PDS, it has the capacity to boost agriculture and reduce hunger and malnutrition by enhancing economic growth [65]. However, the NFSA fails to support environmental sustainability which is vital for the economy and long-term sustenance of the agricultural sector [64,65]. As shown in Figure 2, the NFSA is a double-edged sword which requires careful monitoring and modifications in order to achieve its full potential [65].

While each determinant present in the concept map is vital for safeguarding food security in India, the key driver that influences most of the factors is policy change. Policy amendments have the capacity to establish food security within the country by regulating the barriers and enablers that affect the operational efficiency of the PDS [21,45,54,56]. Positive steps towards the eradication of hunger, malnutrition, childhood mortality, and environmental sustainability can be attained with effective policies [44,66]. The economy of a country, including the pricing strategies, is also influenced by policy reforms and thus affects subsidised rates which are vital for the effective penetration of the PDS [21]. The concept map thus helps to understand that policy changes coupled with reforms in PDS and NFSA are crucial for India to achieve food security for its inhabitants.

## 5. Discussion

This review examined 23 studies to investigate the role of the PDS in addressing food insecurity in India. The key finding of this review is that, while the PDS has been strengthened over recent years, particularly through efforts to target those most in need, more work remains, particularly around transparency and accountability [67,68,69,70].

### 5.1. Failure of PDS

The PDS is the largest food distribution network in the world [20,71,72], and its effective and efficient functioning is an essential component of any response to food insecurity. However, inefficiencies, miss-targeting, and corruption mean that there remain a large number of food insecure people in India. This review has found two key reasons for the failure of PDS in addressing food security: (1) problems with targeting; and (2) problems of diversion and corruption.

#### 5.1.1. Targeting

In 1997, the PDS was restructured, shifting from a universal system, where all Indians, in principle, were eligible to receive a food subsidy, to a system that targeted those most in need. This targeting had two main purposes: (1) to bring down the ever increasing costs of the system; and (2) to provide more food to those in need [73]. Under this change BPL households continued to receive subsidised foods, while subsidies for APL households were phased out [54]. BPL households were identified via household income, compared against an absolute income line. However, households with any assets (such as televisions, fans, two or four wheeled vehicles, or land) were classified as APL under this change, and despite owning such goods, many of these APL households were food insecure, and with the removal of rations were unable to purchase sufficient goods [21,45,74]. The problem of targeting is compounded by the lack of good quality regular data; no regular official estimates of the actual income of households are conducted, with many households BPL not classified as such, and some BPL households not holding ration cards [73]. These problems with targeting limit the usefulness of the PDS in acting as an effective food safety network [58,75], and are further exacerbated by the existence of illegal cards [76]. 

#### 5.1.2. Diversion and Corruption

Leakage and diversion continue to limit the efficiency of the PDS [77]. Whole India data have shown mixed results over the past two decades, with figures from 2007–2008 showing a 44 percent diversion rate for grain, down from 55 percent three years earlier. While these figures reflect a decrease in losses from the system, there remains a significant amount of food displaced in the system [78]. These diversions of commodities, intended for the PDS, into the general market result in shortages for those who rely on subsidised rations [73]. Targeting has not resolved problems with leakage and diversion, some suggest that it has in fact made it worse [79]. Dual pricing introduced through the TPDS is seen by some as an incentive for stakeholders to divert commodities into the open market where they can command a higher price [80]. These diversions and leakages coupled with the inefficiency in monitoring partly due to the decentralised operation increase the likelihood of ingrained, and ongoing corruption [80].

### 5.2. Mechanisms to Strengthen the PDS

While there are many challenges with the working of the PDS, it has the potential to play an important role in addressing food security in India. This review has identified two mechanisms to reinforce efficiency into the functioning of the PDS: (1) the National Food Security Act, 2013; and (2) tracking and electronic governance.

#### 5.2.1. The National Food Security Act

The introduction of the NFSA in 2013 was a constructive step towards strengthening the PDS. Representing a shift from a traditional welfare approach, to an approach underpinned by the acceptance of the human right to food and adequate nutrition, the NFSA formalises the aim of the PDS to provide subsidised food grains to over 800 million people, or approximately two thirds of India’s population. The broad nature of the NSFA allowed for a number of existing food security measures to be entered into law. The NSFA is underpinned by a life cycle approach, that is, it considers the nutritional requirements of the population across all age groups [81], and it includes both universal aspects, available to all Indians, such as the Midday Meal Scheme and the Integrated Child Development Services Scheme, while retaining PDS targeting. Under the NFSA, 75 percent of the rural and 50 percent of the urban population are entitled to 5 kg food grains per month at Rs 3, Rs 2, and Rs 1 for a kg of rice, wheat, and millet, respectively (100 Rupees (Rs) is equal to 1.39 USD). 

In addition to the formalisation of a number of pre-existing entitlements, the NFSA aimed to reinforce the role of the states in the coordination of the PDS, as well as improving transparency and accountability [26]. With empowerment of women and the vulnerable sections of society among the key objectives of the NFSA, monitoring measures to address issues of corruption, diversion and leakages through better partnerships between the central and state governments are also highlighted [26,28].

While the NFSA has every potential to be a “game changer” to strengthen the agricultural industry and the economy of the country, the ability of the NFSA to have a sustained effect on nutrition is questionable [10,65]. Problems with identifying those households in need have not been resolved by the NSFA, and there remains problems with illegal cards [82]. Finally, the expanding need of food grain associated with the NFSA may be detrimental to the environmental sustainability as it demands increased fertiliser, water, and land use, which if unchecked, may lead to land, air, and water pollution [65]. 

#### 5.2.2. Tracking and Electronic Governance

The role of information and communications technology has the potential to be a critical element of success if endorsed and implemented. With the dissemination of digitalisation into the public sector, computerisation can improve the operation of PDS reducing some leakage [83]. It can aid in the identification of beneficiaries and reduce inclusion and exclusion errors associated with targeting while increasing transparency and accountability [56]. Other technology currently trialed in some areas is the application of global positioning system in tracking the food supply chain. This approach works by ensuring that goods are scanned in and out at all points of the supply chain, and has so far shown a reduction in corruption, leakages, and diversion [57,76], and has also shown that goods provided to consumers are higher after the implementation of the system [84]. 

#### 5.2.3. Food Insecurity in India in a Global Context

India is not alone in seeking a range of measures to combat food insecurity. China, like India has a growing economy and a large population. Despite economic growth in China over recent years, like India, China is home to one of the largest populations of hungry people [85]. While India relies on the PDS to mitigate food insecurity, China has focused significant attention on programs that seek to redistribute wealth, and non-food based social security. China is also experiencing a shift in diet patterns, a shift that is having an impact on agricultural production and on the use of land [86]. Likewise, the food insecurity situation in Brazil is undergoing a transformation in the agricultural sector. Like the populations in China and India, the people of Brazil have an increasingly global diet, forcing a change in agricultural patterns. This transition, however, is being supported by the government, alongside cash transfers and school meal programs as an avenue to address increasing food insecurity, with initial indications suggesting some success [87,88]. Given the very large populations in need in these countries, there is unlikely to be a single solution that will work within or across countries. What is important going forward, is that complete and comprehensive data are collected to effectively evaluate these programs. 

### 5.3. Limitations

Several limitations within the literature studied needs to be acknowledged. The limited information on recruitment, data collection, and analysis across the 23 articles included in the review makes comparison difficult and makes any attempt at meta-analysis impossible. The use of secondary data by most of the studies can also affect the quality of the results as it could be outdated or inaccurate among many other pitfalls leading to measurement errors or bias [21,44,54,56,64,65,66,89].

While the authors have attempted to ensure a comprehensive search strategy and methodology for undertaking the literature review, additional articles may have been missed. The complexity of the research topic including the multi-sectoral and multi-dimensional nature of food security has also posed limitations on the literature review. There is also a possibility of relevant grey literature, or literature not available to the public having been missed. Publications in languages other than English may also have been missed.

## 6. Conclusions

The findings of the review suggest a failure at the policy level. The PDS is a cornerstone of government policy responding to nutrition and food security. However, it is riddled with inefficiencies that decrease its capacity to effectively distribute food to those in need. One positive response has been the implementation of the NFSA in 2013, which has strengthened the PDS by providing statutory backing. As evidenced by the review, policy reforms targeted at improving the operational efficiencies and sustainability aspect of the PDS and NFSA are vital for its success. The PDS may not be able to eliminate the issue of malnutrition and childhood morbidity or mortality in India, but it can reduce the levels of hunger in India if implemented effectively. Integrating the PDS with other interventions including those that will increase transparency and accountability may increase its potential to realise every citizen’s right to nutritious food while propagating good health.

The review is the first of its kind to examine the effectiveness of PDS in addressing food insecurity in India. The study also observed the lack of published research around PDS, NFSA and food security in India. This raises the possibility of missing out on existing interventions that have the potential to improve the food distribution network in India. Overall, the review brings out the need for more dedicated research in the field of food security in India which is vital for identifying best practice solutions that will improve the efficacy and operation of the PDS.

## Figures and Tables

**Table 1:** Summary of included studies.

	References	Study Location	Study Design	Methods
1	[49]	Chhattisgarh	Qualitative	Semi-structured interviews and observation.
2	[50]	Kashmir	Qualitative	Household interviews.
3	[52]	Maharashtra, West Bengal	Quantitative	Secondary data analysis. National Sample Survey (NSS) data, round 43.
4	[53]	Tamil Nadu	Quantitative	Household interviews.
5	[42]	India	Quantitative	Secondary data analysis. Bulletin on Food Statistics (1973–1989).
6	[47]	Uttar Pradesh	Mixed	Primary and secondary data analysis: Household interviews. (primary data), NSS, round 68 (secondary data).
7	[48]	Uttar Pradesh	Mixed	Structured survey And interviews
8	[45]	Andhra Pradesh, Maharashtra, Rajasthan	Mixed	Household interviews.
9	[54]	Rajasthan	Quantitative	Secondary data analysis. (NSS, round 55).
10	[21]	Andhra Pradesh, Chhattisgarh, Odisha Tamil Nadu, West Bengal	Quantitative	Secondary data analysis (NSS, rounds 61–66).
11	[55]	Chhattisgarh	Quantitative	Secondary data analysis (NSS, rounds 55 and 61).
12	[43]	India	Quantitative	Secondary data analysis (NSS, rounds 38, 50, 61 and 66).
13	[56]	Bihar	Quantitative	Secondary data analysis (NSS, rounds 50, 61, 66 and 68).
14	[57]	Odisha	Quantitative	Secondary data analysis (NSS, rounds 50, 61and 68).
15	[58]	Assam, Mizoram, Uttar Pradesh, Bihar, Chhattisgarh, Rajasthan, Delhi, Jharkhand, Kerala, Madhya Pradesh, Maharashtra Uttarakhand	Quantitative	Semi structured interviews
16	[59]	Andhra Pradesh, Odisha	Quantitative	Structured household interviews.
17	[60]	Kerala	Quantitative	Secondary data analysis (NSS, round 61).
18	[61]	Kerala	Quantitative	Secondary data analysis (NSS, round 61).
19	[51]	Odisha	Qualitative	Household interviews.
20	[46]	Bihar	Mixed	Structured village interviews.
21	[62]	Kerala	Quantitative	Structured household interviews.
22	[44]	India	Quantitative	Secondary data analysis (NSS, round 55).
23	[63]	Andhra Pradesh	Quantitative	Secondary data analysis (1992–1993 Indian National Family Health Survey and NSS, round 50).
